# Class I and II Histone Deacetylase Inhibitors Differentially Regulate Thermogenic Gene Expression in Brown Adipocytes

**DOI:** 10.1038/s41598-018-31560-w

**Published:** 2018-08-30

**Authors:** Anubama Rajan, Hang Shi, Bingzhong Xue

**Affiliations:** Center for Obesity Reversal, Department of Biology, Georgia State University, Atlanta, GA 30303 USA

## Abstract

Class I histone deacetylase inhibitors (HDACis) enhance whole body energy expenditure and attenuate high fat diet-induced insulin resistance. However, it is not clear whether this is exerted directly through activating brown fat thermogenesis. Here, we find that pan-HDACi TSA exerts paradoxical effects on brown fat gene expression, as it inhibits the expression of *Ucp1, Pparγ and Prdm16* in brown adipocytes, while promoting the expression of other brown fat-specific genes such as *Pgc1α*, *Pgc1β*, *Acox1* and *Cidea*. Further studies indicate that class I HDACi MS-275 significantly increases; whereas class II HDACi MC-1568 markedly reduces, the expression of *Ucp1* and other brown fat-specific genes in treated brown adipocytes. ChIP assay reveals an enhanced H3 acetylation at the *Pgc1α* promoter in MS-275-treated brown adipocytes; whereas the effect of MC-1568 is associated with up-regulation of retinoblastoma protein (*Rb*) and an enhanced acetylation of H3K27 at the *Rb* promoter. Loss of function studies indicate that *Pgc1α* up-regulation largely mediates the stimulatory effect of class I HDACis on the thermogenic program, whereas up-regulation of *Rb* may be responsible for the inhibitory effect of class II HDACis. Thus, our data suggest that class I and II HDACis have differential effects on brown fat thermogenic gene expression.

## Introduction

Obesity has become a world-wide epidemic problem, and is an independent risk factor for a panel of metabolic diseases, including insulin resistance/type 2 diabetes, dyslipidemia, cardiovascular diseases, and certain types of cancer^1^. Therefore, it is important to better understand the mechanisms regulating energy homeostasis and to identify pathways that can intervene the etiology of obesity.

Obesity results from chronic imbalance between energy intake and energy expenditure^1^. While white adipose tissue (WAT) stores energy in the form of triglyceride, brown adipose tissue (BAT) dissipates energy through adaptive thermogenesis due to the unique expression of uncoupling protein 1 (UCP1) in the inner membrane of mitochondria^1^. Activation of brown adipocyte thermogenesis by pharmacological or genetic approaches increases energy expenditure and alleviates obesity in rodents^2–8^. Recent discovery of functional brown and beige adipocytes in humans suggest that increasing brown fat thermogenic function may be a novel approach in treating obesity^9–11^.

Most of the current studies investigating the mechanisms underlying the regulation of brown fat functions focus on cellular signaling pathways; less is known about the epigenetic mechanisms in this process. Epigenetic modifications, including histone acetylation, regulate gene expression by changing the chromatin structures without altering the actual DNA sequences^12,13^. Histone acetylation levels are maintained by dynamic actions of histone acetyltransferases (HATs) responsible for acetylation and histone deacetylases (HDACs) responsible for deacetylation^14^. The HDAC family can be divided into four major classes, each of which consists of different HDAC members: class I (including HDAC 1, 2, 3 and 8), class II (including HDAC 4, 5, 6, 7, 9, 10), class III (including SIRTs 1–7) and class IV (HDAC 11). Class I, II and IV HDACs are a group of enzymes that catalyze the removal of acetyl groups from lysine residues in histones and cellular proteins, whereas class III HDACs (Sirt1–Sirt7) form a distinct family of NAD-dependent deacetylases that can be inhibited by nicotinamide^14^. Recent studies have suggested a key role for HDACs in the regulation of body weight, energy metabolism and insulin sensitivity^15^. For instance, Gao *et al*. reported that pan-HDAC inhibitors (pan HDACis) sodium butyrate and TSA decrease body weight and improve insulin sensitivity via increasing energy expenditure in diet-induced obese (DIO) mice^16^. Further studies demonstrated that the beneficial effects of pan HDACis on energy metabolism and insulin sensitivity might be mediated through the inhibition of class I HDACs, since class I HDACi MS-275 mimics the beneficial effects of pan HDACis in DIO mice^17^. However, it is not clear whether class I HDACis enhance overall energy expenditure through activating brown fat thermogenesis, an integral part of total energy expenditure. In addition, our previous data suggested a paradoxical effect of the pan-HDACis TSA and SAHA on brown adipocyte gene expression^18^. While TSA and SAHA stimulated certain brown adipocyte thermogenic gene expression, including *Pgc1α* and *Acox1*, they profoundly inhibited the expression of other brown adipocyte-specific genes, including *Ucp1*, *Pparγ* and otopetrin 1 (*Otop1*)^18^.

Thus, to further clarify the differential effects of various HDACis on the regulation of brown adipocyte thermogenic program, we treated brown adipocytes with pan-HDACi TSA and SAHA, class I HDACi MS-275, and class II HDACi MC-1568, and measured the expression of thermogenic genes. We further determined the molecular pathways underlying the alteration of thermogenic gene expression by HDACi treatment. We assessed the histone acetylation status at the specific gene promoters and determined whether these genes mediated the effects of these HDACis via the loss of function approach with SiRNA knockdown.

## Results

### Pan-HDAC inhibitors exhibit a mixed effect on the expression of brown fat genes

**Figure 1:**
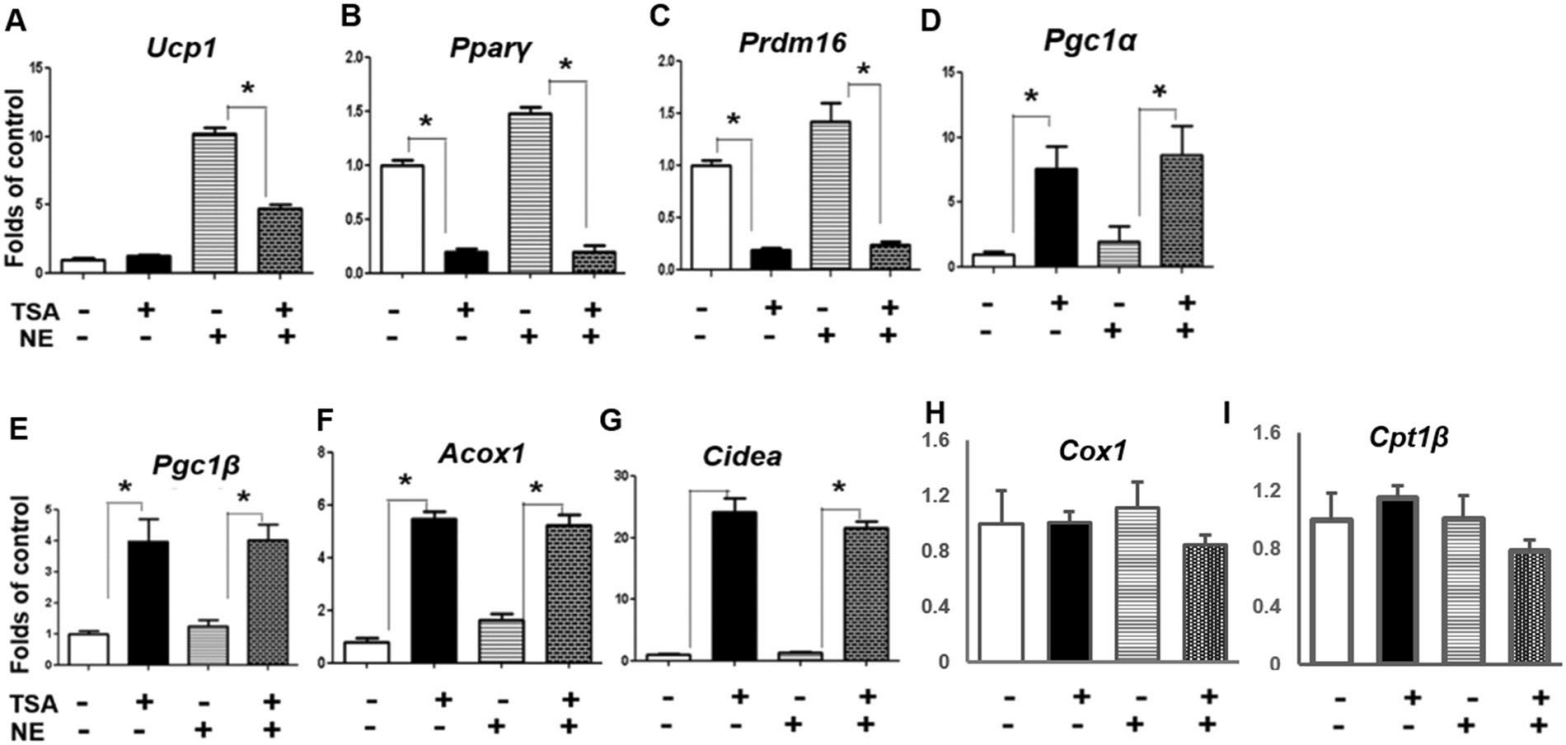
Pan-HDAC inhibitor TSA exhibits a mixed effect on the expression of brown fat genes. TSA inhibits the expression of brown fat genes *Ucp1* (**A**), *Pparγ* (**B**), and *Prdm16* (**C**), while increasing others including *Pgc1α* (**D**), *Pgc1β* (**E**), *Acox1* (**F**), and *Cidea* (**G**) in HIB-1B cells. TSA has no effects on *Cox1* (**H**) and *Cpt1b* (**I**) expression. Cells were pre-treated with TSA (500 nM) for 30 min, followed by stimulation with norepinephrine (NE, 1 µM) for 4 hours. Brown adipocyte mRNA was measured by quantitative RT-PCR as described in Methods. All data are expressed as mean ± SEM, n = 4–6; *p < 0.05.

To determine the effect of HDACis in the regulation of brown adipocyte thermogenic program, we first tested pan-HDACis TSA and SAHA in the brown adipocyte cell line HIB-1B cells. In our previous study, we found that TSA (5, 50 and 500 nM) dose-dependently inhibited the expression of *Ucp1, Pparγ*, PR domain-containing protein 16 *(Prdm16)* and *Otop1*, while stimulating *Pgc1α* expression in BAT1 brown adipocyte cell line^18^. In addition, SAHA (50 nM, 500 nM and 5 μM) also dose-dependently inhibited the expression of *Ucp1, Pparγ*, and *Otop1* while stimulating *Pgc1α* expression in BAT1 cells^18^. The dose ranges of TSA and SAHA have been commonly used and shown to inhibit both class I and class II HDAC activities in cell culture systems^17,19–22^. Thus, we chose to use 500 nM TSA and 5 μM SAHA in our current study to achieve maximum effects. Similar to our previous findings^18^, TSA treatment resulted in a significant decrease in the expression of some brown fat genes such as *Ucp1*, *Pparγ*, and *Prdm16* mRNA (Fig. 1A–C). SAHA, another pan HDACi, also showed a similar inhibitory effect on *Ucp1* expression (Suppl. Figure 1). In contrast, we observed a robust increase in other brown fat genes including *Pgc1α*, *Pgc1β*, *Acox1*, *Cidea* in HIB-1B cells treated with TSA (Fig. 1D–G); whereas TSA had no effects on the expression of cytochrome c oxidase subunit I (*Cox1*) and carnitine palmitoyltransferase 1b, muscle (*Cpt1b*) (Fig. 1H,I). Moreover, TSA displayed a similar effect in inhibiting certain brown fat genes such as *Ucp1*, *Pparγ*, *Prdm16*, type 2 deiodinase (*Dio2*), elongation of very long chain fatty acids (FEN1/Elo2, SUR4/Elo3, yeast)-like 3 (*Elovl3*) and *Otop1*, while stimulating others such as *Pgc1α*, *Pgc1β*, and *Acox1* in another brown adipocyte cell line BAT1 (Suppl. Figure 2); whereas TSA had no effect on the expression of *Cox1* and *Cpt1*b (Suppl. Figure 2). Our data suggest that indiscriminate inhibition of all HDACs by pan HDACis results in a mixed effect on the expression of brown fat genes and that different classes of HDACs may have a different effect in this regard.

### Class I HDAC inhibitor MS-275 promotes the expression of brown fat genes

**Figure 2:**
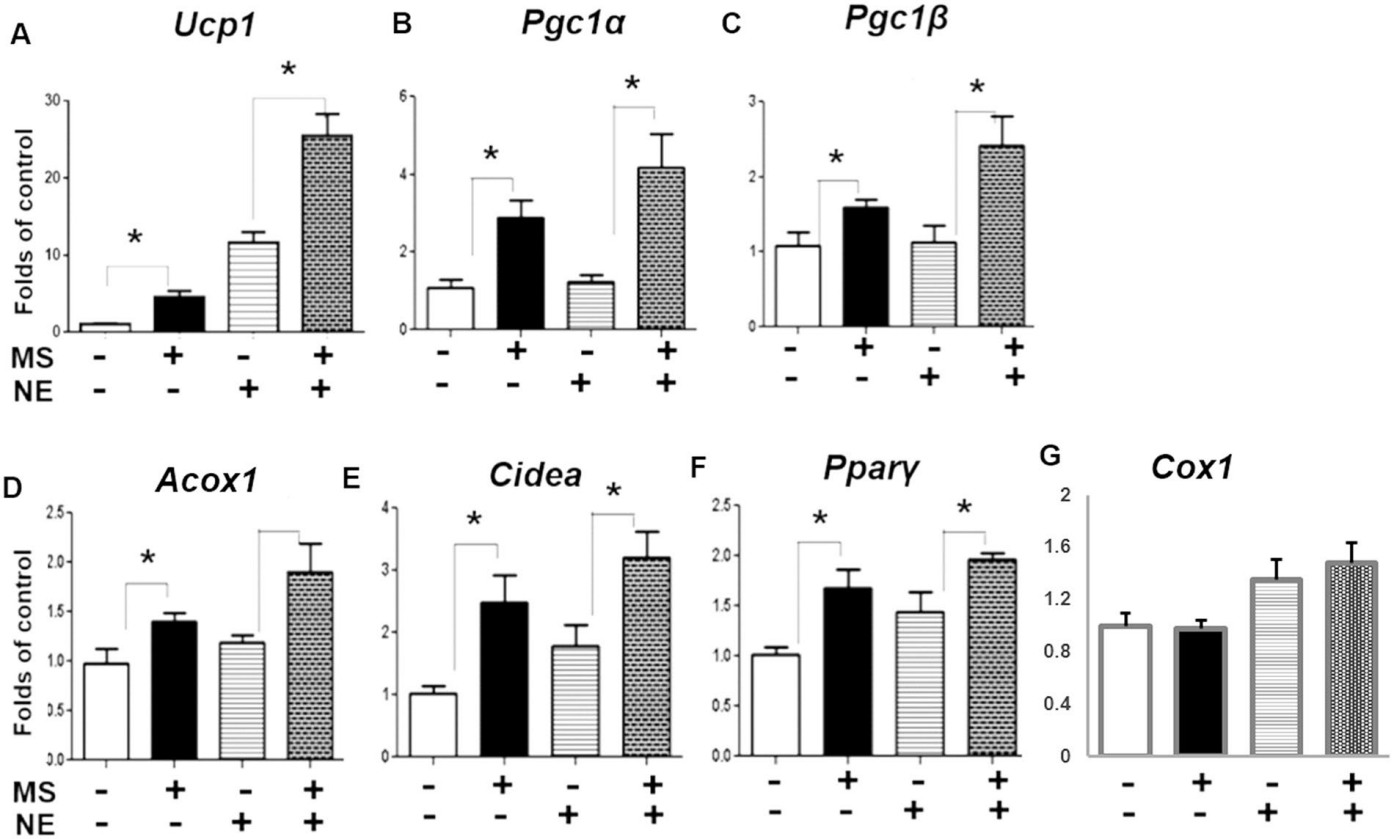
Class I HDAC inhibitor MS-275 promotes the expression of brown fat genes *Ucp1* (**A**), *Pgc1α* (**B**), *Pgc1β* (**C**), *Acox1* (**D**), *Cidea* (**E**) and *Pparγ* (**F**), but has no effect on *Cox1* expression (**G**) in HIB-1B cells. Cells were pre-treated with MS-275 (MS, 5 µM) for 30 min, followed by stimulation with norepinephrine (NE, 1 µM) for 4 hours. Brown fat mRNA was measured by quantitative RT-PCR as described in Methods. All data are expressed as mean ± SEM, n = 3–6; *p < 0.05.

**Figure 3:**
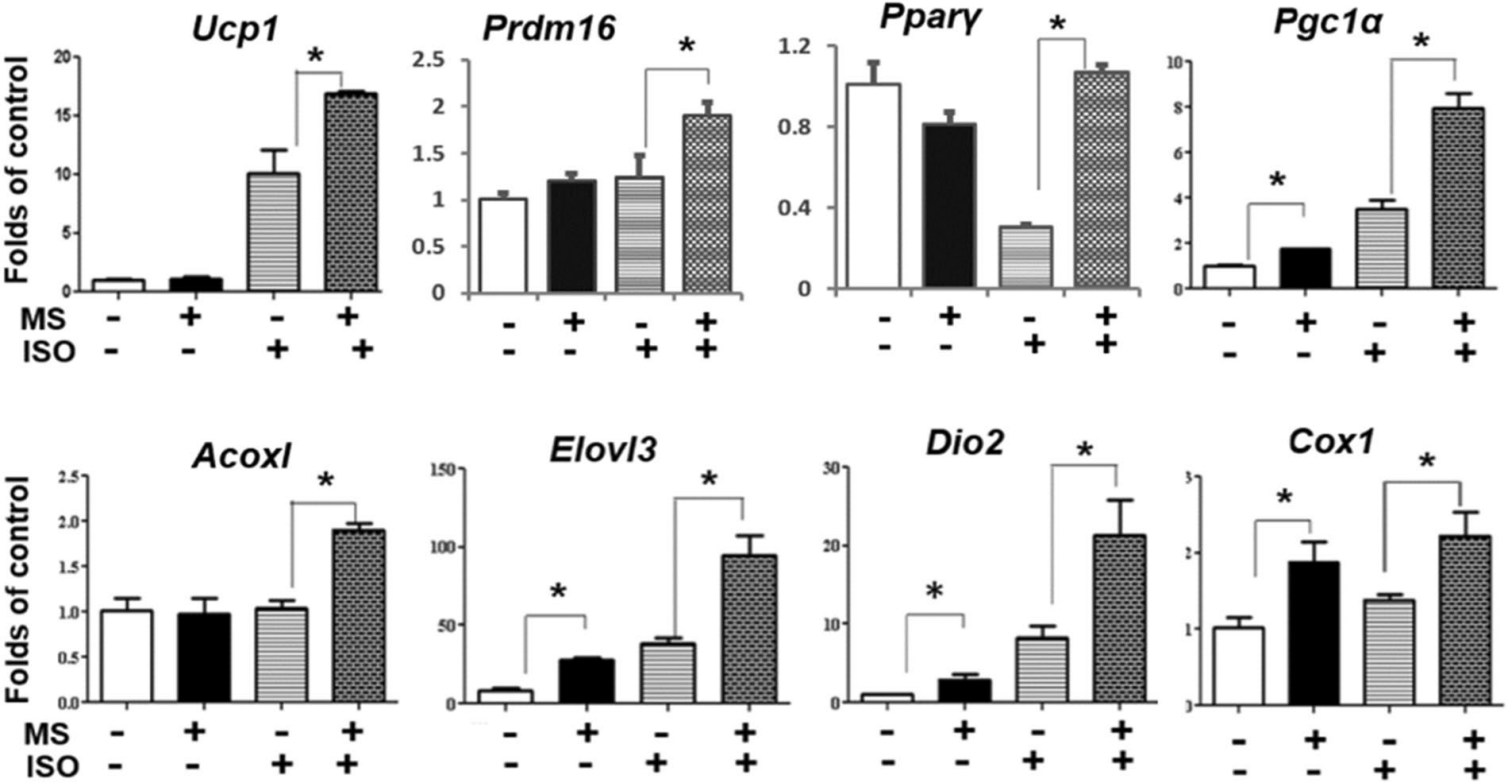
Class I HDAC inhibitor MS-275 promotes the expression of brown fat genes *Ucp1*, *Prdm16, Pparγ*, *Pgc1α*, *Acox1*, *Elovl3*, *Dio2* and *Cox1* in BAT1 cells. BAT1 cells were pre-treated with MS-275 (MS, 5 µM) for 30 min, followed by stimulation with isoproterenol (ISO, 1 µM) for 3 hours. Brown adipocyte mRNA was measured by quantitative RT-PCR as described in Methods. All data are expressed as mean ± SEM, n = 3; *p < 0.05.

To resolve the paradoxical effect of pan HDACis in the expression of brown fat genes, we employed experiments to distinguish the effect of class I and class II HDACis by treating the brown adipocytes with individual class HDACis. In our previous study, we found that MS-275 dose-dependently (50 nM, 500 nM and 5 μM) stimulated brown fat thermogenic gene expression, including *Ucp1, Pparγ, Elovl3* and *Pgc1α* in BAT1 cells^18^. These MS-275 doses (up to 5 μM) have been used in the literature to preferentially inhibit class I HDACs^17,23,24^. Thus, we chose to use 5 μM MS-275 in current study for maximal effects. We found that treatment of HIB-1B cells with class I HDACi MS-275 significantly enhanced basal and isoproterenol-induced brown fat gene expression including *Ucp1*, *Pgc1α*, *Pgc1β*, *Acox1*, *Cidea*, and *Pparγ*, whereas MS-275 had no effect on *Cox1* expression (Fig. 2). A similar stimulation of thermogenic genes by MS-275 was observed in another brown adipocyte BAT1 cells, including *Ucp1*, *Prdm16*, *Pparγ*, *Pgc1α*, *Acox1*, *Elovl3*, *Dio2* and *Cox1* (Fig. 3). Interestingly, consistent with the stimulatory effect on the thermogenic program in brown adipocytes, MS-275 treatment also caused a marked increase in *Ucp1* mRNA expression in 3T3-L1 adipocytes, a white adipocyte cell line, in a time course experiment (Suppl. Figure 3).

**Figure 4:**
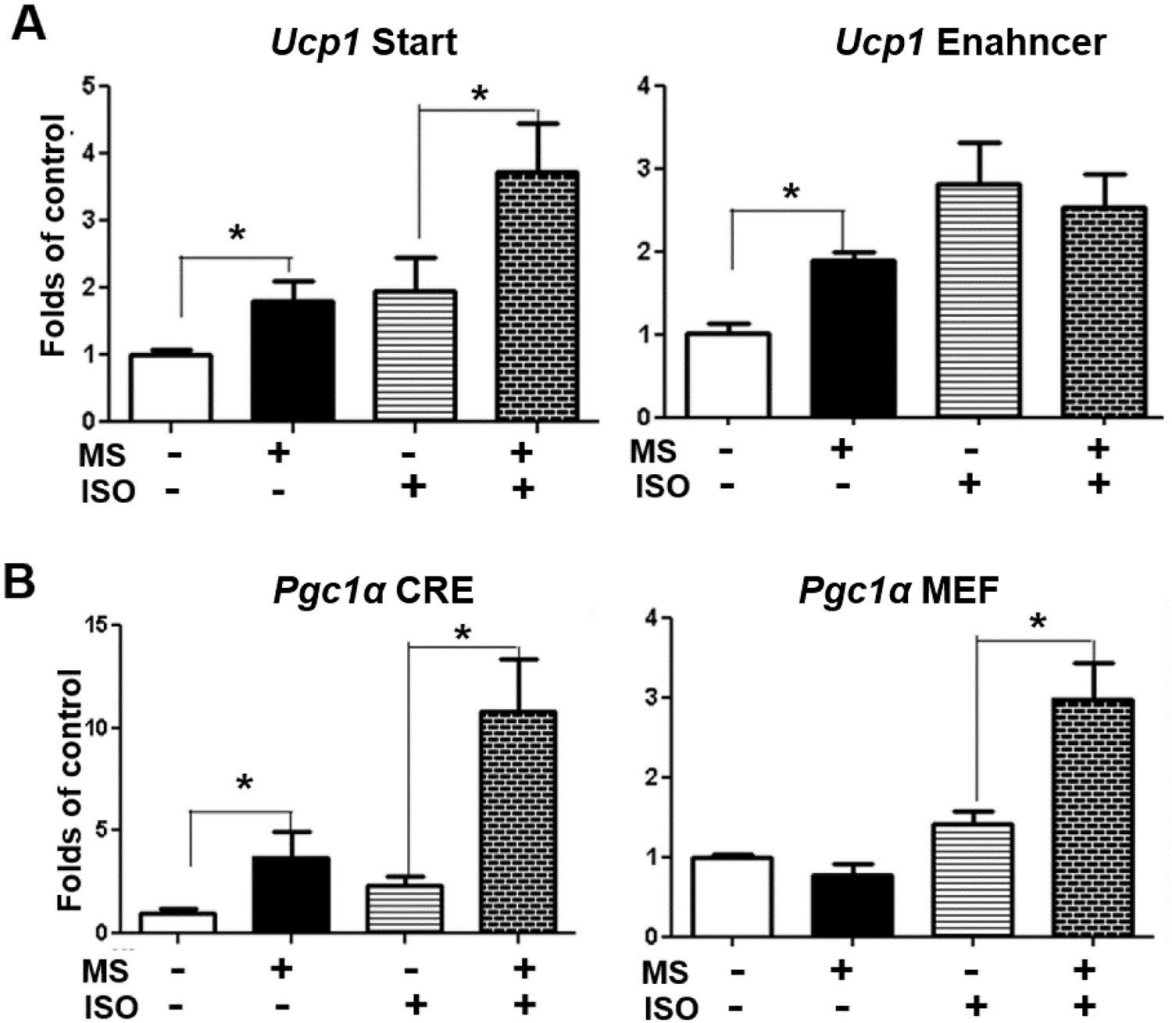
MS-275 enhances H3 acetylation at the *Ucp1* promoter (**A**) and *Pgc1α* promoter (**B**). BAT1 cells were pre-treated with MS-275 (MS, 5 µM) for 30 min, followed by stimulation with isoproterenol (ISO, 1 µM) for 3 hours. The H3 acetylation at the promoters was evaluated by ChIP assays followed by SYBR Green quantitative PCR as described in Methods. All data are expressed as mean ± SEM, n = 4; *p < 0.05. Start: transcription start site; CRE: cAMP response element; MEF: myocyte enhancer factor.

We think that inhibiting class I HDAC by MS-275 may enhance acetylation on certain histone lysine sites, which subsequently results in DNA unwinding and likely turns on gene expression responsible for the thermogenic program in brown adipocytes. To explore the mechanism underlying the stimulatory effect of class I HDACis in brown fat genes, we focused on histone acetylation at the promoter regions of *Pgc1α* and *Ucp1* genes. UCP1 is the mitochondrial uncoupler in BAT for adaptive thermogenesis^25,26^, whereas PGC1α is the master regulator of brown fat thermogenic program^27,28^. The *Ucp1* promoter contains an upstream enhancer element that is responsible for the brown fat-specific expression of *Ucp1*^29,30^. Moreover, *Pgc1α* expression is upregulated by β adrenergic activation via the downstream signaling protein kinase A (PKA)/cAMP response element (CRE) binding protein (CREB) pathway^31,32^, which binds to the CRE *cis*-element on the *Pgc1α* promoter^33^, and by myocyte enhancer factor 2 (MEF2) binding to its cis-element MEF binding site^34^. We therefore examined the histone H3 acetylation (H3ac) at the transcription start and enhancer regions of the *Ucp1* promoter and the key *cis*-elements of CRE and MEF of the *Pgc1α* promoter, respectively. Our ChIP assays revealed an enhanced H3ac at both transcription start and enhancer regions of the *Ucp1* promoter in brown adipocytes treated with class I HDACi MS-275 (Fig. 4A). Activation of β adrenergic signaling by isoproterenol increased H3ac at the transcription start site, which was further increased by MS-275, although MS-275 had no effect on isoproterenol-stimulated H3ac at the enhancer region (Fig. 4A). In consistence, MS-275 exhibited a similar stimulatory effect on H3ac at the *Pgc1α* promoter. MS-275 treatment up-regulated H3ac at the CRE site of the *Pgc1α* promoter, although no effect was observed at the MEF site (Fig. 4B). Nonetheless, isoproterenol-stimulated H3ac was further enhanced by MS-275 at the *cis*-elements of both CRE and MEF (Fig. 4B).

To determine the role of PGC1α in mediating the effect of MS-275 on *Ucp1* expression, we knocked down *Pgc1α* by SiRNA in brown adipocytes followed by MS-275 treatment. The *Pgc1α* mRNA expression was reduced by more than 70% in the knockdown cells (Suppl. Figure 4A). *Pgc1α* knockdown significantly reduced basal and isoproterenol-stimulated *Ucp1* mRNA (Suppl. Figure 4B). *Pgc1α* inactivation also substantially prevented the enhanced *Ucp1* expression caused by MS-275 treatment (Suppl. Figure 4B).

### Class II HDAC inhibitor MC-1568 inhibits Ucp1 expression

**Figure 5:**
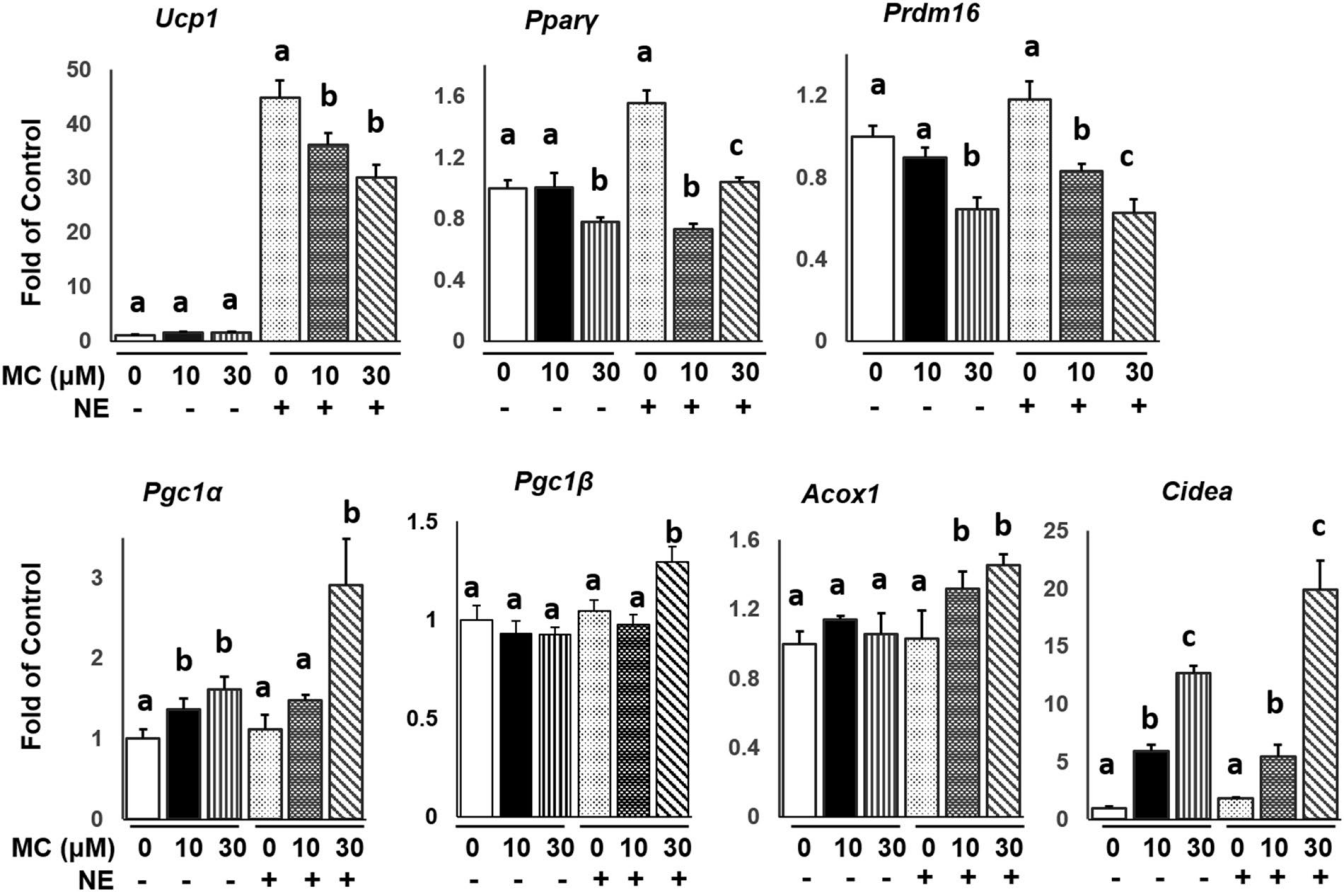
Class II HDAC inhibitor MC-1568 inhibits the expression of *Ucp1*, *Pparγ* and *Prdm16*, but stimulates the expression of *Pgc1α*, *Pgc1β*, *Acox1* and *Cidea* in brown adipocytes. HIB-1B cells were pre-treated with MC-1568 (10 or 30 µM) for 30 min, followed by stimulation with norepinephrine (NE, 1 µM) for 4 hours. Gene expression was measured by quantitative RT-PCR as described in Methods. All data are expressed as mean ± SEM, n = 3–6; Bars with different letters are significantly different from each other in control groups (without NE) or NE-treated groups.

We next determined the effect of class II HDACi MC-1568 in brown adipocytes. We found that MC-1568 treatment at 10 and 30 μM inhibited basal- and/or NE-induced thermogenic gene expression, including *Ucp1, Pparγ and Prdm16* in HIB-1B cells (Fig. 5). These MC-1568’s inhibitory effects are similar to pan HDAC inhibitors TSA and SAHA’s effects (Fig. 1, Suppl. Figures 1 and 2), but are opposite to that of class I HDACi MS-275 (Figs 2 and 3). In contrast, MC-1568 treatment (10 and 30 μM) in HIB-1B cells significantly enhanced basal- and/or NE-induced certain brown adipocyte-specific thermogenic gene expression, including *Pgc1α*, *Pgc1β, Acox1* and *Cidea*, with a slightly more potent effect exerted at 30 μM (Fig. 5). These MC-1568’s stimulatory effects are mostly similar to those of pan HDAC inhibitors TSA and SAHA’s, and the class I HDACi MS-275’s effects (Figs 1–3 and Suppl. Figures 1–2). In contrast, MC-1568 had no effect on Cox1 expression (Suppl. Figure 5). Similar effects were also observed in BAT1 brown adipocytes (Suppl. Figure 6). Our data suggest that both class I and II HDACis activate certain groups of thermogenic gene expression, including *Pgc1α, Pgc1β*, *Acox1* and *Cidea;* however, they differ significantly in their effects on other thermogenic gene expression. Whereas class I HDACis activate gene expression of *Ucp1*, *Pparγ* and *Prdm16*; class II HDACis significantly inhibit these gene expression.

**Figure 6:**
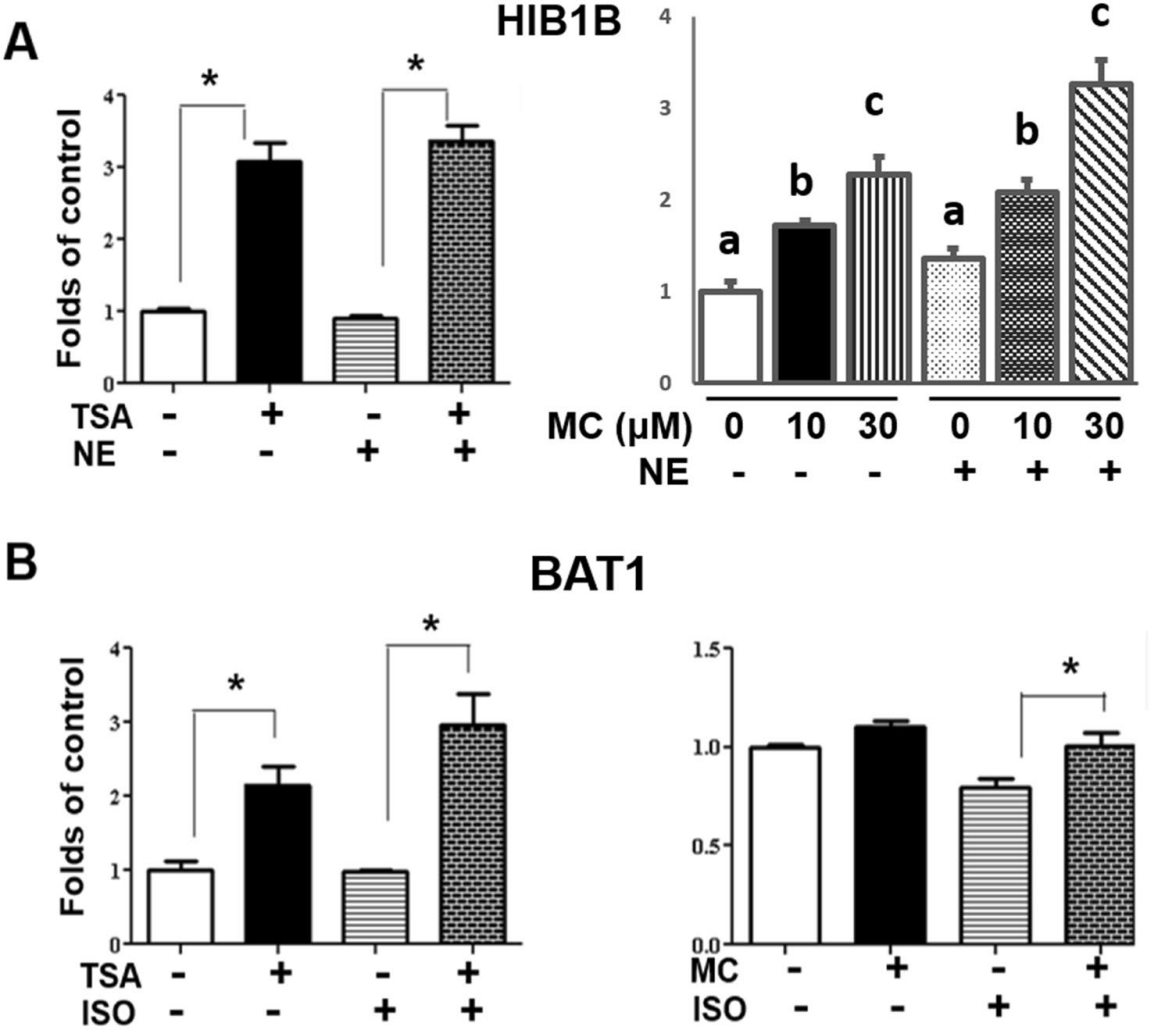
Pan HDAC inhibitor TSA or class II HDAC inhibitor MC-1568 increases the expression of *Rb* in brown adipocytes. HIB-1B or BAT1 cells were pre-treated with TSA (500 nM) or MC-1568 (MC, 10 or 30 µM) for 30 min, followed by stimulation with norepinephrine (NE, 1 µM) for 4 hours (HIB-1B) or isoproterenol (ISO, 1 µM) for 3 hours (BAT1). *Rb* mRNA was measured by quantitative RT-PCR as described in Methods. All data are expressed as mean ± SEM, n = 3–6; *p < 0.05. Bars with different letters are significantly different from each other in control groups (without NE) or NE-treated groups.

We sought to determine the mechanism underlying the inhibitory effect of class II HDACis on *Ucp1* and other thermogenic gene expression. Since inhibition of HDACs by either pan HDACis or class II HDACis results in enhanced histone acetylation that likely turns on gene expression, we reasoned that certain transcriptional repressors that inhibit brown fat thermogenic gene expression might be the potential targets of class II HDACi MC-1568, activation of which might be responsible for the inhibitory effect of MC-1568 on *Ucp1* expression. Among the transcriptional repressors we surveyed (data not shown), retinoblastoma protein (*Rb*), which has been shown to repress *Ucp1* expression^35^, was up-regulated in both HIB-1B and BAT1 brown adipocytes by TSA and MC-1568, respectively (Fig. 6). However, MS-275 that stimulates the expression of *Ucp1* and other brown fat genes, had no effect or even caused a slight inhibition on *Rb* expression in brown adipocytes (Suppl. Figure 7). These data suggest that up-regulation of *Rb* by TSA and MC-1568 may mediate the inhibitory effect of these compounds on *Ucp1* and other thermogenic gene expression.

**Figure 7:**
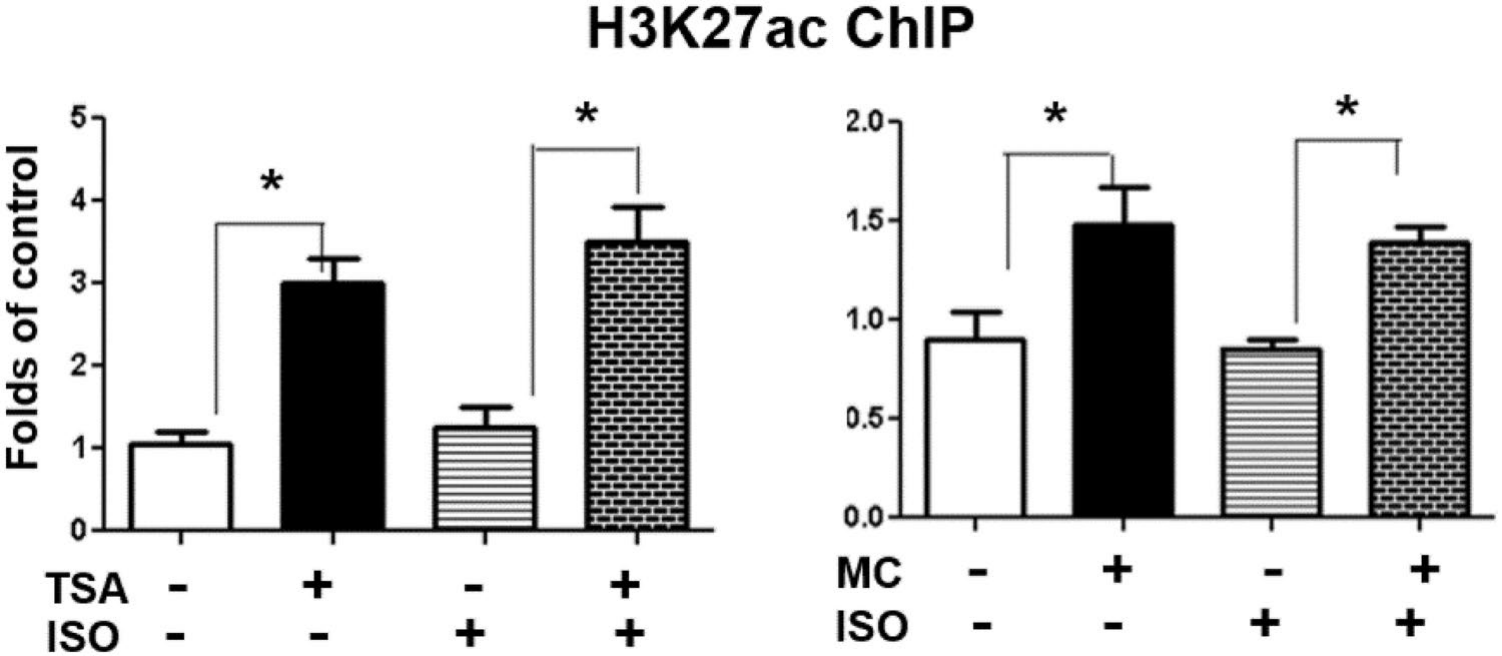
TSA or MC-1568 enhances H3K27 acetylation at the *Rb* promoter. BAT1 cells were pre-treated with TSA (500 nM) or MC-1568 (MC, 30 µM) for 30 min, followed by stimulation with isoproterenol (ISO, 1 µM) for 3 hours. The H3K27 acetylation at the *Rb* promoter was evaluated by ChIP assays followed by SYBR Green quantitative PCR as described in Methods. All data are expressed as mean ± SEM, n = 4; *p < 0.05.

To investigate the mechanism underlying the up-regulation of *Rb* expression by MC-1568, we assessed the histone acetylation status at the *Rb* promoter, focusing on histone H3 lysine 27 acetylation (H3K27ac), a transcriptional active mark for gene expression^36^. Using ChIP assays, we found that treating BAT1 cells with TSA or MC-1568 significantly enhanced H3K27ac at the *Rb* promoter (Fig. 7).

**Figure 8:**
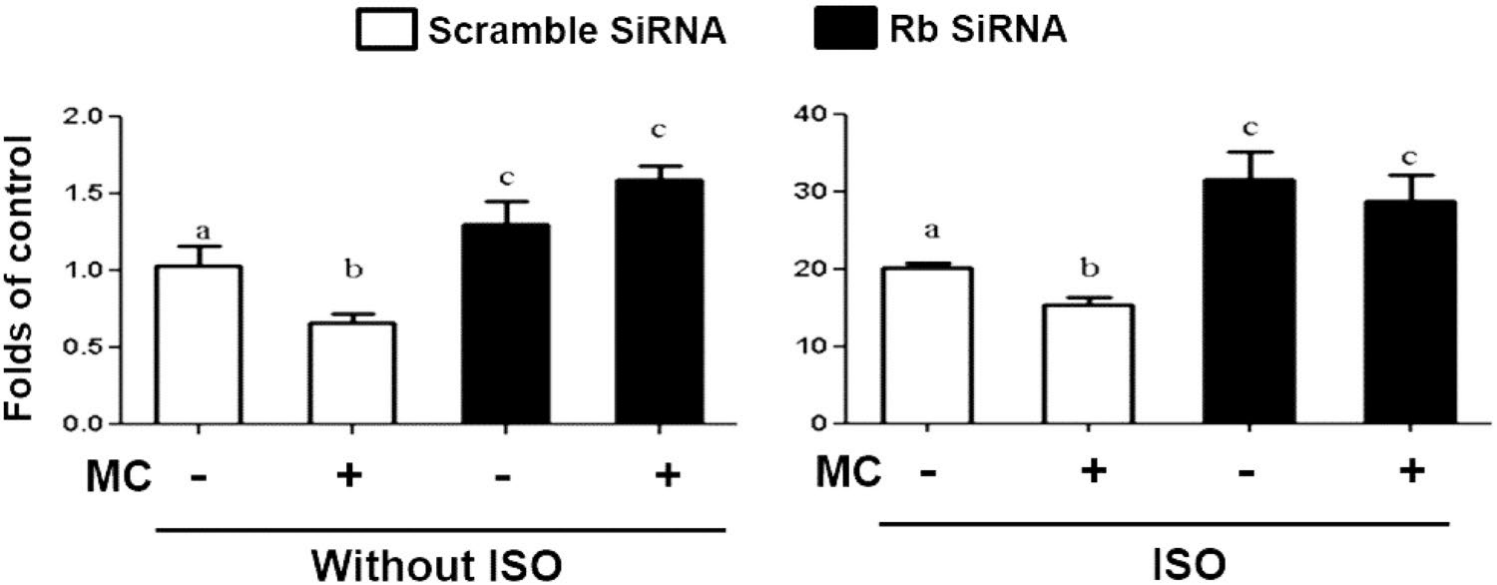
*Rb* knockdown substantially prevents the inhibitory effect of MC-1568 on Ucp1 expression. BAT1 cells were pre-treated with MC-1568 (MC, 30 µM) for 30 min, followed by stimulation with isoproterenol (ISO, 1 µM) for 3 hours. *Ucp1* mRNA was measured by quantitative RT-PCR as described in Methods. All data are expressed as mean ± SEM, n = 4; *p < 0.05.

To determine the role of *Rb* in mediating the inhibitory effect of TSA and MC-1568 on *Ucp1* expression, we knocked down *Rb* by SiRNA in BAT1 brown adipocytes. The *Rb* mRNA expression was decreased by 70% in the knockdown cells (Suppl. Figure 8). As expected, MC-1568 treatment down-regulated *Ucp1* expression at both basal and isoproterenol-stimulated levels, which was substantially prevented by *Rb* knockdown (Fig. 8). Similar results were observed in *Rb* knockdown BAT1 cells treated with TSA (Suppl. Figure 9), which displayed a similar inhibitory effect on *Ucp1* expression as MC-1568.

Finally, immunoblotting analysis showed that TSA, MS-275 and MC-1568 stimulated acetylated histone H3 (acetyl-H3) levels in HIB-1B cells as expected (Suppl. Figure 10).

## Discussion

In this study, we aimed to evaluate the effect of HDAC inhibitors (HDACis) in the regulation of brown fat gene expression. The plausibility of this study derives from several prior findings in the investigation of epigenetics and brown fat thermogenesis in obesity. First, epigenetic regulations have emerged as important mechanisms in the development of obesity and may serve as promising therapeutic targets for the prevention and treatment of obesity^37–40^. However, much remains unknown about epigenetic regulation of the brown fat thermogenesis, an integral part of overall energy expenditure. Second, recent studies have suggested that inhibiting HDACs by HDACis have therapeutic potentials in diet-induced obesity and insulin resistance^15^. For example, pan HDAC inhibitors sodium butyrate and trichostatin A (TSA) have been shown to reduce body weight and ameliorate insulin resistance in DIO mice^16^. An attempt has been made to determine which class of the HDACs might mediate the beneficial effect of pan HDACis. Galmozzi *et al*. have demonstrated that class I HDACis recapitulated the beneficial effect of the pan HDACis, while class II HDACis had no effect^17^. However, it is not clear whether this was exerted directly through activating brown fat thermogenesis by HDACis. Because the data were derived from animal studies where treatment of HDACis in animals might exert effect in multiple organs, it is difficult to distinguish whether HDACi directly regulates brown fat function and energy metabolism.

Here we first tested the effect of pan HDACis in the regulation of brown adipocyte thermogenic program. We found that pan HDACi TSA down-regulated the expression of certain brown fat genes, notably *Ucp1, Pparγ* and *Prdm16*, while up-regulating others including *Pgc1α* and *Pgc1β*. The data demonstrate a mixed effect of pan HDACis in the regulation of brown fat thermogenic genes. Therefore, although pan HDACis can seemingly promote oxidative metabolism in brown and white fat as shown by previous study^17^, the inhibitory effect of pan HDACis on *Ucp1* expression as we discovered here does not likely contribute to the enhanced overall energy expenditure. The paradoxical role of pan HDACis made us think that inhibition of class I or class II HDAC may result in discrepant or even contradictory effect on the brown fat thermogenic function. We therefore further tested the effect of class I or class II HDACis in the regulation of brown fat thermogenic gene expression. Indeed, we found that class I HDAC inhibitor MS-275 promotes the expression of brown fat thermogenic genes including *Ucp1*, *Pparγ* and *Prdm16*, which is opposite to the effect of pan HDACis TSA and SAHA on these gene expression patterns. In contrast, class II HDACi MC-1568 significantly suppressed these brown adipocyte gene expression, similar to the effects of the pan-HDACis TSA and SAHA on these gene expression.

Thus, we found that whereas all HDACis tested in the present study stimulated certain brown adipocyte-specific gene expression, including *Pgc1α, Pgc1β* and *Acox1*, their effects on other brown adipocyte gene expression are profoundly different. Whereas the pan-HDACi TSA and the class II HDACi MC-1568 significantly inhibit the expression of *Ucp1, Pparγ* and *Prdm16*, the class I HDACi MS-275 significantly stimulates these gene expression. It is currently unknown how MS-275 exerts different effects on the expression of *Ucp1, Pparγ* and *Prdm16* than that of TSA and MC-1568. Although HDACs have relatively low substrate specificity by themselves, a single enzyme being capable of deacetylating multiple sites within histones^12^, the enzyme specificity of HDACs may be determined by other factors. For example, class I HDACs are found primarily in the nucleus and ubiquitously expressed throughout all human tissues; whereas Class II enzymes can be found in both the nucleus and cytoplasm^14^. In addition, different HDACs are found to be associated with multiple distinct complexes^12^. For instance, HDAC1 is found together with HDAC2 within the NuRD, Sin3a and Co-REST complexes; whereas HDAC 3 is found within the nuclear receptor co-repressor (N-coR) and Silencing Mediator for Retinoid and Thyroid receptors (SMRT) repressor complex^12^. On the other hand, the unique structural features in each HDAC may eventually determine the target specificity of each HDACs and their associated complexes. HDAC6 possesses a zinc-finger domain for ubiquitin binding, allowing the recognition and transport of ubiquitylated proteins and controlling polyubiquitin-chain turnover; whereas chromodomains, plant homeodomain-linked (PHD) fingers and other modification-specific modules are present in different HDAC complexes^41^. Thus, these unique features of each HDAC may determine their differential target specificity and influence their physiological function.

To determine the pathway mediating the effect of class I HDACis, we assessed the histone acetylation at the promoter regions of *Pgc1α*, a transcriptional regulator of brown fat thermogenic program. We found an enhanced H3 acetylation on the two *cis*-elements CRE and MEF sites that are critical for *Pgc1α* transcription. DNA unwinding might be ensued by the histone acetylation, which can subsequently increase accessibility for transcription factors such as CREB to bind to its response element at the *Pgc1α* promoter and activate its gene transcription. We think the up-regulation of *Pgc1α* might be largely responsible for the enhanced expression of *Ucp1* by class I HDACi MS-275, although it is noteworthy that MS-275 can directly promote H3 acetylation on the CRE and start regions at the *Ucp1* promoter. Loss of *Pgc1α* by SiRNA knockdown largely prevented the stimulatory effect of MS-275 on *Ucp1* expression, suggesting the key role of *Pgc1α* in mediating MS-275’s action on *Ucp1* expression.

Since inhibition of HDAC by HDACis supposedly increases histone acetylation that may eventually turn on gene expression, we reasoned that the suppression of *Ucp1* expression by MC-1568 or the pan inhibitor TSA may be due to up-regulation of transcriptional repressors by these HDAC inhibitors. Through a survey of repressor expression, we narrowed down to *Rb*, a well-known negative regulator of Ucp1 expression^35,42^. Since both TSA and MC-1568 promoted *Rb* expression while MS-275 had no effect, we therefore reckoned that the inhibition of HDACs by TSA or MC-1568 activates *Rb* expression that mediates the suppression of *Ucp1* expression. To determine the importance of the up-regulation of *Rb* expression in mediating TSA/MC-1568’s inhibition of *Ucp1* expression, we examined the epigenetic regulation of the *Rb* promoter by these compounds and performed a loss of function experiment by knocking down *Rb*. We found that TSA or MC-1568 enhanced H3K27ac, a transcriptional active mark, on the *Rb* promoter, which may lead to enhanced *Rb* expression, while *Rb* knockdown largely prevented TSA or MC-1568’s inhibitory effect on *Ucp1* expression. The data demonstrate that *Rb* mediates the suppression of *Ucp1* expression by TSA and MC-1568 in brown adipocytes. This may also explain the paradoxical role of the pan HDACi TSA in the regulation of the brown fat thermogenic program. It is due to TSA’s ability to induce both *Rb* and *Pgc1α* through histone acetylation, which subsequently represses *Ucp1* but up-regulates other brown fat genes.

Emerging evidence has suggested epigenetic regulation as an important mechanism underlying obesity and energy metabolism^37–40^. For instance, *Ucp1* expression is regulated by DNA methylation at the *Ucp1* promoter^43^. Moreover, the histone demethylase JHDM2a promotes *Ucp1* expression via decreasing H3K9 methylation, a transcriptional repressive mark. This is physiologically important in energy metabolism, since mice with genetic deletion of JHDM2a are prone to diet-induced obesity^44^. Therefore, epigenetic mechanisms underlying thermogenic program may serve as promising therapeutic targets for the prevention and treatment of obesity. Our data strongly indicate that inhibition of class I HDACs by specific inhibitors could be one of these therapeutic strategies. Class I HDACs have four family members including HDAC 1, 2, 3, and 8^15^. Our previous data demonstrated that knocking down *Hdac1* (and to a lesser extent, *Hdac8*) mimicked the effect of MS-275 and activated brown fat thermogenic gene expression^18^. Thus, *Hdac1* (and possibly *Hdac8*) may be the major HDACs that mediating the effects of class I HDACis on brown fat thermogenic function.

In contrast, we find that class II HDACi MC-1568 differentially regulates brown fat thermogenic gene expression in that it inhibits certain brown fat-specific gene expression including *Ucp1, Pparγ* and *Prdm16* while promoting other brown fat thermogenic gene expression, such as *Pgc1α, Pgc1β* and *AcoxI*. The gene expression pattern regulated by the class II HDACi MC-1568 is mostly similar to that of the pan-HDACis but is significantly different from that of class I HDACi MS-275. Thus, further whole genome RNA-sequencing analysis in brown adipocytes treated with pan-HDACis, class I HDACis or class II HDACis will help to thoroughly understand the differential effects of these different classes of HDACis in the regulation of brown adipocyte function.

Class II HDACs include HDACs 4, 5, 6, 7, 9 and 10^15^. In our previous studies, we found that while knocking down the class II HDACs 5 and 9 increased *Ucp1* expression, knocking down HDACs 6, 7 and 10 slightly reduced *Ucp1* expression in HIB-1B brown adipocytes^18^. Thus, further studies will be needed to identify the specific (or a combination of) class II HDACs that mediate MC-1568’s effects.

In summary, we demonstrate that class I and II HDACis have differential effects on the regulation of brown fat thermogenic gene expression. Class I HDAC inhibitor MS-275 up-regulates *Pgc1α* expression by increasing H3ac at the Pgc1α promoter, leading to the promotion of thermogenic gene expression in brown adipocytes. In contrast, class II inhibitor MC-1568 stimulates *Rb* expression by enhancing H3K27ac at the *Rb* promoter, resulting in an inhibition of *Ucp1* expression. Our data indicate that class I HDAC inhibitors may directly promote brown fat thermogenesis, which may contribute to the beneficial effect of HDACis in obesity and its associated disorders, and that pharmacological inhibition of class I HDACs may serve as a promising therapeutic target for the prevention and treatment of obesity.

## Methods

### Reagents

All HDAC inhibitors, MS-275, MC-1568 and Trichostatin (TSA) were obtained from selleck Chemicals (Houston, TX).

### Cell culture

All cells used in this study were cultured at 37 °C with 5% CO_2_. Immortalized brown preadipocytes BAT1 (kindly provided by Dr. Patrick Seale, University of Pennsylvania)^45^ were cultured in DMEM/F12 mixture medium containing 10% fetal bovine serum and 1% Penicillin/Streptomycin. HIB-1B preadipocytes were grown in DMEM medium with the same supplements as BAT1. Brown adipocyte differentiation was induced as described previously^45^. Briefly, brown preadipocytes were grown up to 90% confluence in growth medium and were then induced to differentiation with a differentiation medium containing the growth medium supplemented with 20 nM insulin, 1 nM triiodothyronine (T3), 125 μM indomethacin, 500 μM isobutylmethylxanthine and 0.5 μM dexamethasone. Cells were maintained in the differentiation medium for two days and then switched to a maintenance medium containing the growth medium supplemented with 20 nM insulin and 1 nM T3 only, for 6 days. Brown adipocytes were pre-treated with HDACis for 30 min followed by stimulation with isoproterenol or norepinephrine (NE) for 3–4 hours to induce the thermogenic gene expression.

3T3-L1 preadipocytes were cultured and differentiated as we previously described^46,47^. Briefly, L1 preadipocytes were grown in a DMEM medium containing 10% new born calf serum and 5% penicillin. To induce differentiation, cells were grown to post-confluence for 2 days and then switched to a differentiation medium of DMEM supplemented with 10% fetal bovine serum (FBS), 0.5 mM 3-isobutyl-1-methylxanthine, 1 μM dexamethasone, and 800 nM insulin. Cells were incubated in the differentiation medium for 2 days and then changed to growth medium containing 800 nM insulin only for another 2 days. Subsequently, cells were maintained in DMEM medium containing 10% FBS (without insulin) throughout the adipocyte stage.

### Total RNA Isolation and quantitative RT-PCR

Total RNA was extracted from BAT1/HIB-1B cells using Tri-Reagent kit (Molecular Research Center, Cincinnati, OH) according to the manufacturer’s instruction. The quantitation of the mRNAs of genes of interest was assessed by quantitative real-time RT-PCR (ABI Universal PCR Master Mix, Applied Biosystems, Foster City, CA) using a Stratagene Mx3005p thermocycler (Stratagene, La Jolla, CA) and was normalized by the corresponding cyclophilin mRNA measurement as we previously described^46,47^. Some primer and probe pairs used in the assays were purchased from Applied Biosystems and the primer/probe sequences for other genes are provided in Summplemental Table 1.

### Immunoblotting Analysis for acetyl-H3 and total H3

HIB1B cells were treated with TSA, MS-275 or MC-1568 as indicated, and immunoblotting analysis was performed as we previously described^18^. Briefly, cell samples were homogenized in a modified radioimmune precipitation assay lysis buffer containing 50 mM Tris-HCl, 1 mM EDTA, 1% Nonidet P-40, 0.5% sodium deoxycholate, 150 mM NaCl, 1 mM sodium fluoride, 1 mM phenylmethylsulfonyl fluoride, 1 mM sodium orthovanadate, 1% protease inhibitor mixture (Sigma), and 1% phosphatase inhibitor mixture (Sigma). Cell homogenates were incubated on ice for 45 min to solubilize all proteins, and insoluble portions were removed by centrifugation at 14,000 × g at 4 °C for 15 min. Proteins from whole cell lysates were separated by SDS-PAGE and transferred to polyvinylidene difluoride membranes. The transferred membranes were blocked, washed, and incubated with anti-histone H3 and anti-acetyl-histone H3 (Millipore) antibodies, followed by incubation with Alexa Fluor 680-conjugated secondary antibodies (Life Technologies). The fluorescent signal was visualized with a LI-COR Imager System (LI-COR Biosciences, Lincoln, NE).

### SiRNA Knockdown

The ON-TARGET Mouse [peroxisome proliferator activated receptor γ (PPARγ) coactivator 1α (Pgc1α)] or retinoblastoma protein (Rb) siRNA–SMART pool was purchased from GE Dharmacom (GE Healthcare). HIB-1B cells were reversely transfected with SiRNA to achieve the gene knockdown using a Lipofectamine RNAiMAX Reagent kit (Invitrogen) as we previously described^48,49^. BAT1 cells were transfected with SiRNAs by Amaxa NucleofectorII Electroporator (Lonza) using an Amaxa cell line nucleofector kit L according to manufacturer’s instructions (Lonza) as we previously described^48^.

### Chromatin Immuno-Precipitation (ChIP) assays

ChIP assays were performed using a ChIP assay kit (Upstate, Lake Placid, NY) as we previously described^48,49^. The cells were fixed with 1% formaldehyde, washed and collected in ice cold PBS buffer containing protease inhibitors 1 mM phenylmethylsulfonyl fluoride (PMSF) and 1 µg/ml pepstatin A. After a brief centrifugation, the pellet was suspended in SDS lysis buffer containing 1% SDS, 10 mM EDTA and 50 mM Tris. The cell lysates were sonicated to obtain genomic DNA fragments with the length ranging between 200–1000 bp. These lysates were then centrifuged, and the supernatants of the sample were collected. The supernatants underwent overnight immunoprecipitation, elution, reverse cross-linking, and protease K digestion, according to the manufacturer’s manual. All the antibodies were obtained from Abcam and Millipore. Eluted DNA was analyzed by real-time PCR using SYBR green quantitative PCR (Life Technologies). Primer sequences used for ChIP in this study are provided in Supplemental Table 2.

### Statistics

One-way analysis of variance (ANOVA) and least-significant-difference test were performed to evaluate statistical significance using GraphPad Prism version 5.0. Statistical significance was considered at *p* < 0.05. All data are shown as mean ± standard error of means (SEM).

## Electronic supplementary material

Supplemental Tables, Figures and Figure Legends
