# Supplementary material for: Class I and II Histone Deacetylase Inhibitors Differentially Regulate Thermogenic Gene Expression in Brown Adipocytes
Source: Sci Rep. 2018 Aug 30;8:13072. doi: 10.1038/s41598-018-31560-w (PMC6117331; doi:10.1038/s41598-018-31560-w)
Supplement: Supplementary file 1 — Supplemental Tables, Figures and Figure Legends [file 41598_2018_31560_MOESM1_ESM.pdf]

Supplemental Tables, Figures and Figure Legends

Class I and II Histone Deacetylase Inhibitors Differentially Regulate Thermogenic Gene Expression in Brown Adipocytes

Anubama Rajan, Hang Shi and Bingzhong Xue

Center for Obesity Reversal, Department of Biology, Georgia State University, Atlanta, GA 30303, USA

Supplemental Table 1: Primer sequence for TaqMan primer and probes

Gene	Sequence
UCP1	Forward: CACCTTCCCGCTGGACACT<br>Reverse: CCCTAGGACACCTTTATACCTAATGG<br>Probe: AGCCTGGCCTTCACCTTGGATCTGA
PGC1α	Forward: CATTTGATGCACTGACAGATGGA<br>Reverse: CCGTCAGGCATGGAGGAA<br>Probe: CGTGACCACTGACAACGAGGCC
PGC1β	Forward: AGGAAGCGGCGGGAAA<br>Reverse: CTACAATCTCACCGAACACCTCAA<br>Probe: AGAGATTTCGAATGTATACCACACGGCCTTCA
COX-1	Forward: TTTTCAGGCTTCACCCTAGATGA<br>Reverse: GAAGAATGTTATGTTTACTCCTACGAATATG<br>Probe: CATGAGCAAAAGCCCACTTCGCCA
ACOX-1	Forward: AAGAACTCCAGATAATTGGCACCTA or<br>Reverse: AGTGGTTTCCAAGCCTCGAA<br>Probe: CGGAGATGGGCCACGGAACTCAT
Cyclophilin	Forward: GGTGGAGAGCACCAAGACAGA<br>Reverse: GCCGGAGTCGACAATGATG<br>Probe: ATCCTTCAGTGGCTTGTCCCGGCT

Supplemental Table 2: Primer sequence for ChIP analysis

Gene	Sequence
Ucp1 Enhancer region	Forward: CTCCTCTACAGCGTCACAGAGG<br>Reverse: AGTCTGAGGAAAGGGTTGA
Ucp1 Start region	Forward: CCCACTAGCAGCTCTTTGGA<br>Reverse: CTGTGGAGCAGCTCAAAGGT
Pgc1α MEF2	Forward: GCTCGCTGCATTTCTTTCTT<br>Reverse: CCCCACAGACTCAAAAACCA
Pgc1α CRE	Forward: CAAAGCTGGCTTCAGTCACA<br>Reverse: AAAAGTAGGCTGGGCTGTCA
Rb1 Promoter region	Forward: TACTTGGGTTCGAGTCCTCTGCCAG<br>Reverse: AGTTGGCCGTGTTCATGCG

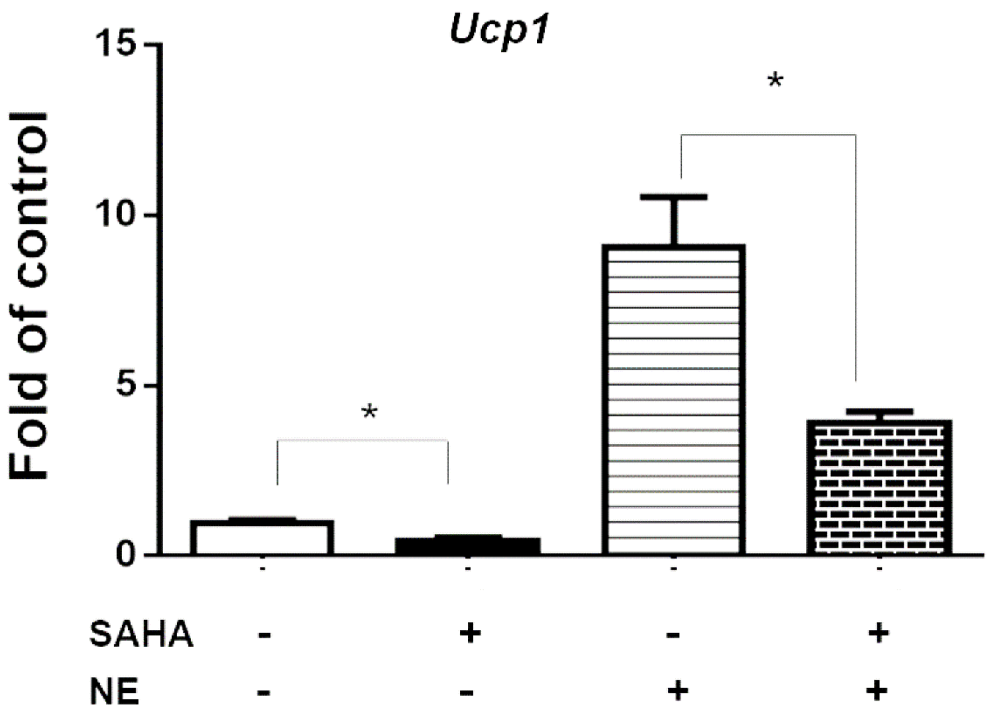

Supplemental figure 1. Pan HDAC inhibitor SAHA inhibits the expression of *Ucp1* in HIB-1B cells. Cells were pre-treated with SAHA (5μM) for 30 min and followed by stimulation with norepinephrine (NE, 1μM) for 4 hours. *Ucp1* mRNA was measured by quantitative RT-PCR as described in Methods. All data are expressed as mean ± SEM, n=4-6; *$p<0.05$.

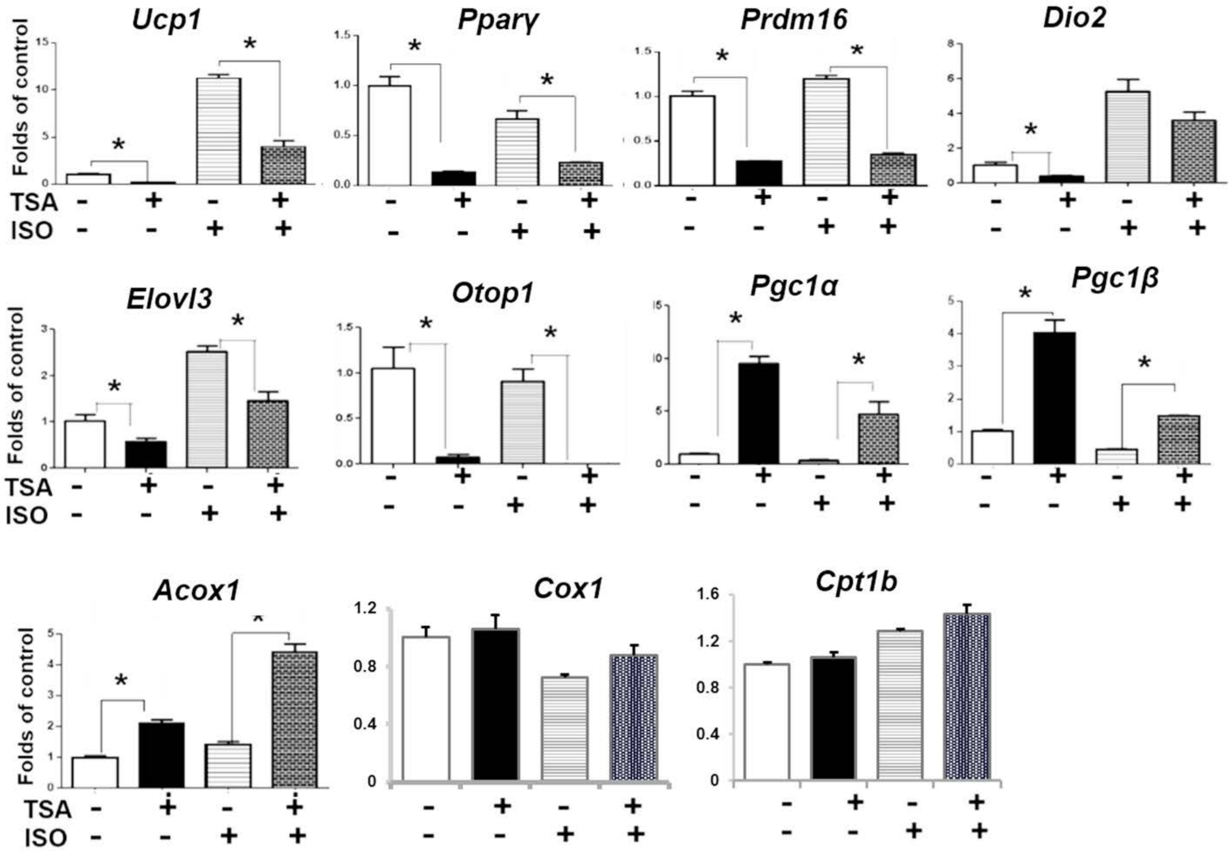

Supplemental figure 2. TSA inhibits the expression of brown fat genes *Ucp1*, *Pparγ*, *Prdm16*, *Dio2*, *Elovl3*, and *Otop1*, promotes others including *Pgc1α*, *Pgc1β*, and *Acox1*, whereas has no effect on *Cox1* and *Cpt1b* expression in BAT1 cells. Cells were pre-treated with TSA (500nM) for 30 min and followed by stimulation with isoproterenol (ISO, 1μM) for 3 hours. Brown adipocyte mRNA was measured by quantitative RT-PCR as described in Methods. All data are expressed as mean ± SEM, n=3; *p<0.05.

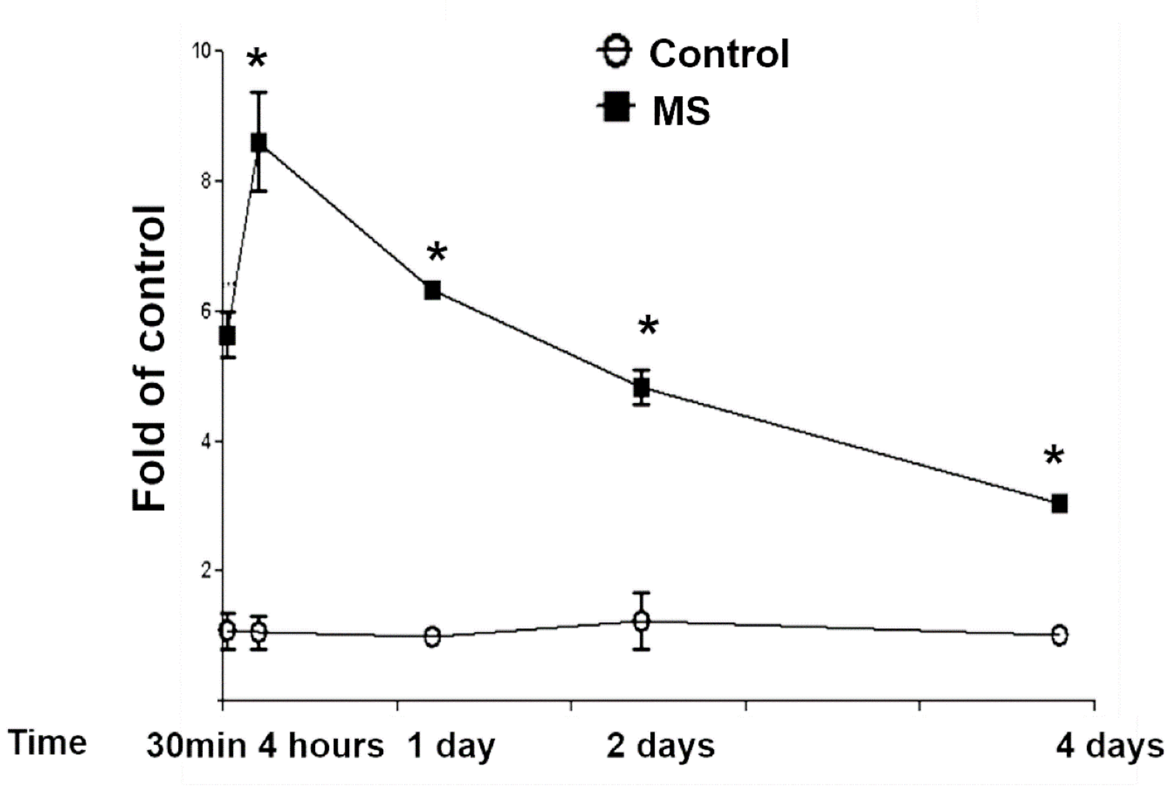

Supplemental figure 3. Class I HDAC inhibitor MS-275 stimulates the expression of *Ucp1* in 3T3-L1 adipocytes. L1 preadipocytes were differentiated into adipocytes, which were then treated with MS-275 (MS, 5µM) in a time course study as indicated in the figure. *Ucp1* mRNA was measured by quantitative RT-PCR as described in Methods. All data are expressed as mean ± SEM, n=3; *$p<0.05$.

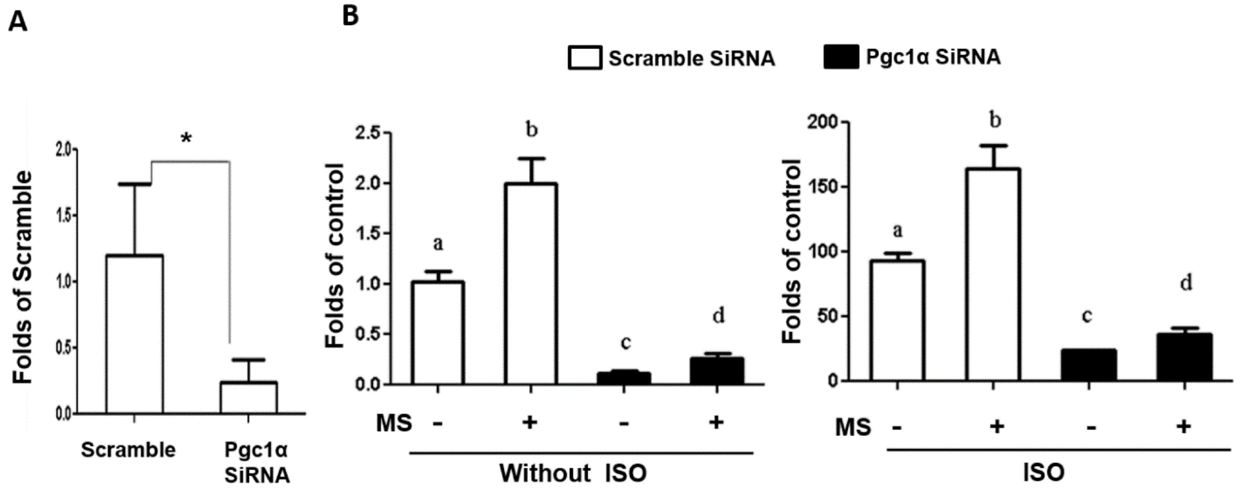

Supplemental figure 4. *Pgc1α* knockdown substantially prevents the stimulatory effect of MS-275 on *Ucp1* expression. BAT1 cells were pre-treated with MS-275 (MS, 5μM) for 30 min and followed by stimulation with isoproterenol (ISO, 1μM) for 3 hours. *Pgc1α* (A) and *Ucp1* (B) RNA was measured by quantitative RT-PCR as described in Methods. All data are expressed as mean ± SEM, n=4; *p<0.05. Bars with different letters are significantly different from each other.

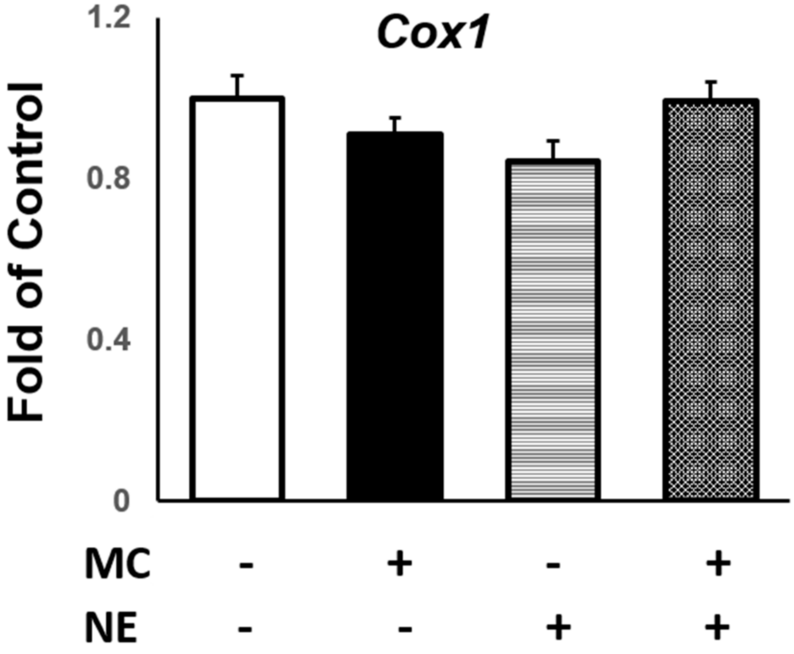

Supplemental figure 5. Class II HDAC inhibitor MC-1568 has no effect on Cox1 expression in HIB1B brown adipocyte cells. Cells were pre-treated with MC-1568 (MC, 30μM) for 30 min and followed by stimulation with norepinephrine (NE, 1μM) for 4 hours. Brown adipocyte mRNA was measured by quantitative RT-PCR as described in Methods. All data are expressed as mean ± SEM, n=4-6.

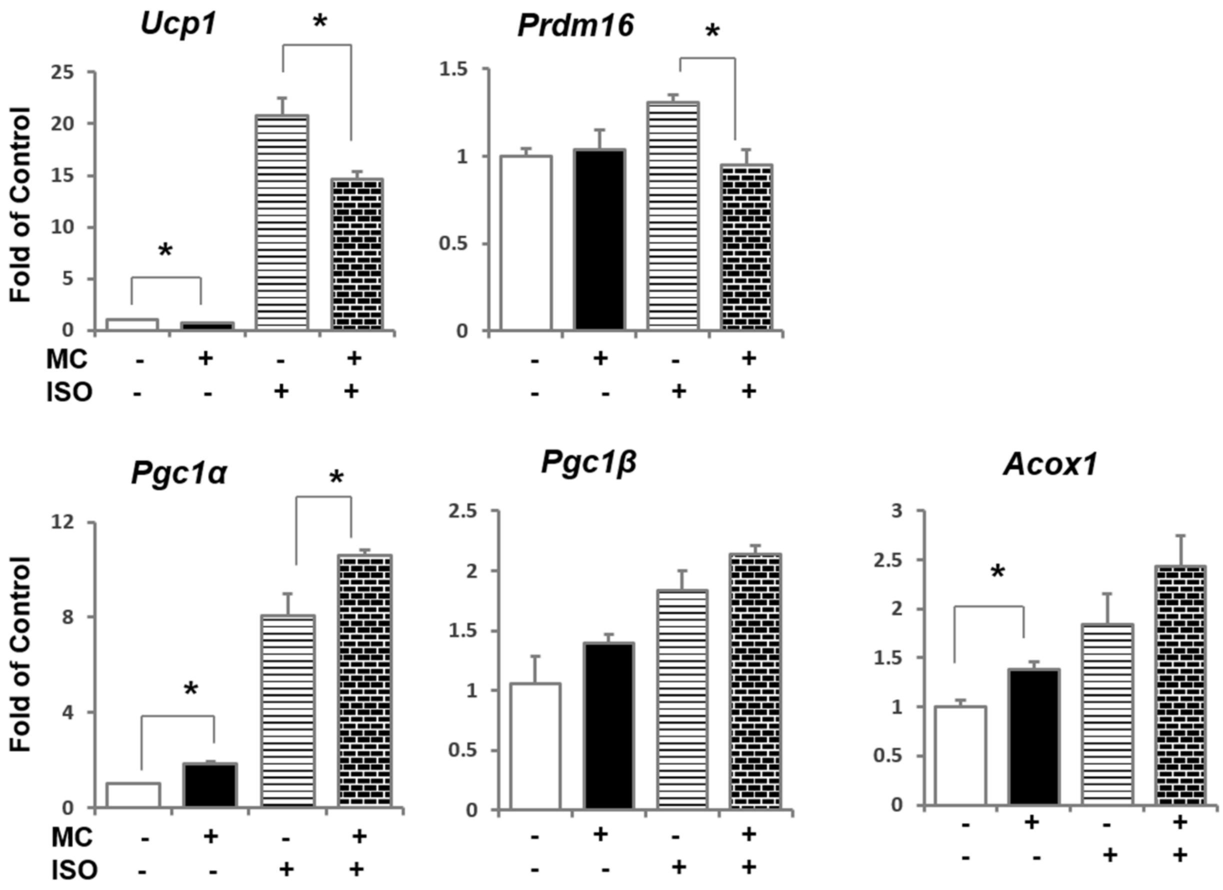

Supplemental figure 6. Class II HDAC inhibitor MC-1568 inhibits the expression of brown fat genes *Ucp1* and *Prdm16*, while promoting (or exerting no effect on) others including *Pgc1α*, *Pgc1β*, and *Acox1* in BAT1 cells. Cells were pre-treated with MC-1568 (MC, 30μM) for 30 min and followed by stimulation with isoproterenol (ISO, 1μM) for 3 hours. Brown adipocyte mRNA was measured by quantitative RT-PCR as described in Methods. All data are expressed as mean ± SEM, n=3; *p<0.05.

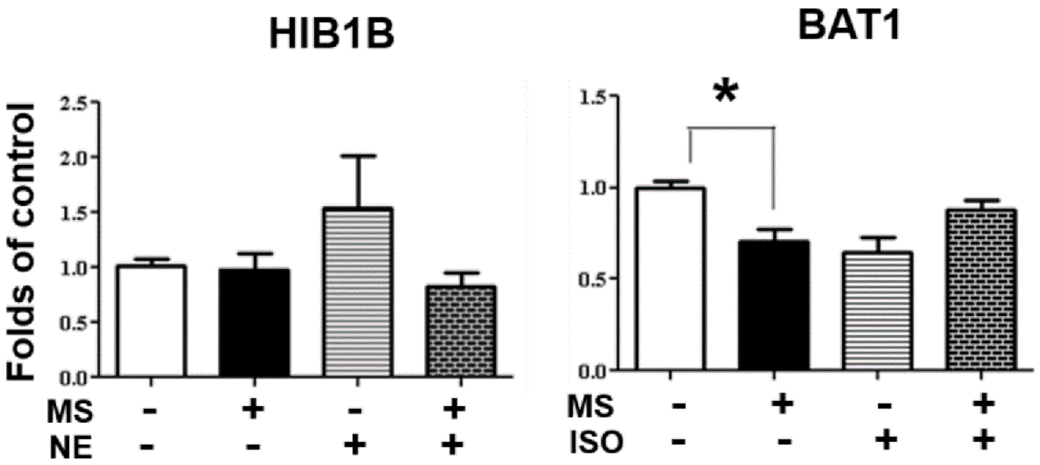

Supplemental figure 7. Class I HDAC inhibitor MS-275 has little effect on *Rb* expression. HIB-1B or BAT1 cells were pre-treated with MS-275 (MS, 5μM) for 30 min and followed by stimulation with norepinephrine (NE, 1μM) for 4 hours (HIB-1B) or isoproterenol (ISO, 1μM) for 3 hours (BAT1).. *Rb* mRNA was measured by quantitative RT-PCR as described in Methods. All data are expressed as mean ± SEM, n=3-6; *p<0.05.

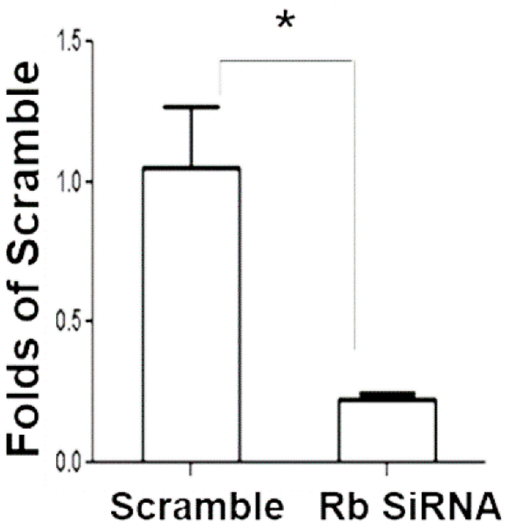

Supplemental figure 8. *Rb* mRNA expression was knocked down by 70% in BAT1 cells. BAT1 cells were reversely transfected with *Rb* SiRNA as described in Materials and Methods. *Rb* mRNA was measured by quantitative RT-PCR as described in Methods. All data are expressed as mean ± SEM, n=4; *$p<0.05$.

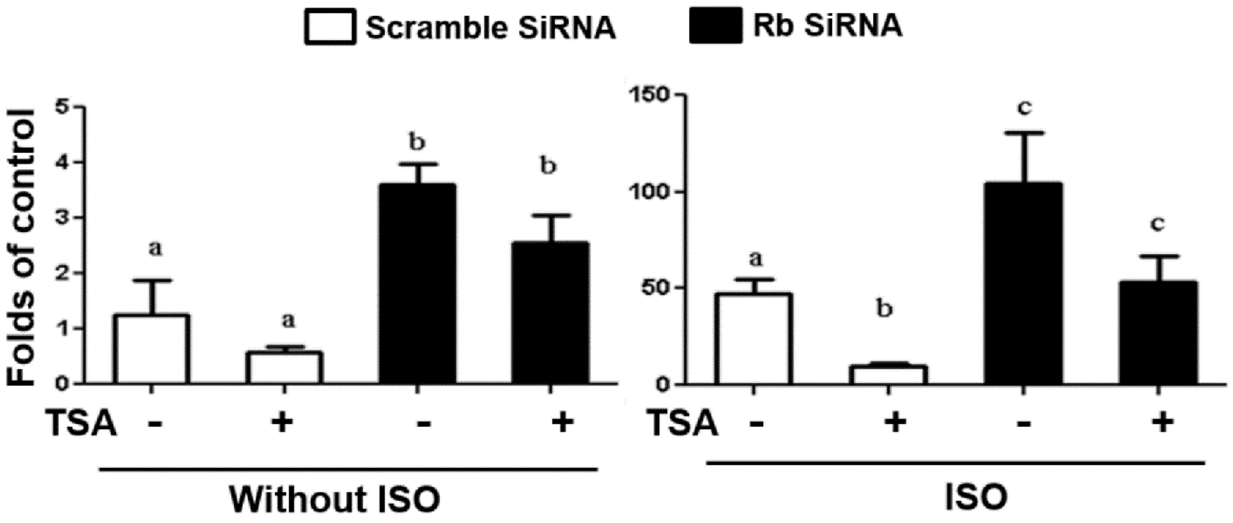

Supplemental figure 9. *Rb* knockdown substantially prevents the inhibitory effect of TSA on *Ucp1* expression. BAT1 cells were pre-treated with TSA (500nM) for 30 min and followed by stimulation with isoproterenol (ISO, 1μM) for 3 hours. *Ucp1* mRNA was measured by quantitative RT-PCR as described in Methods. All data are expressed as mean ± SEM, n=4; *p<0.05.

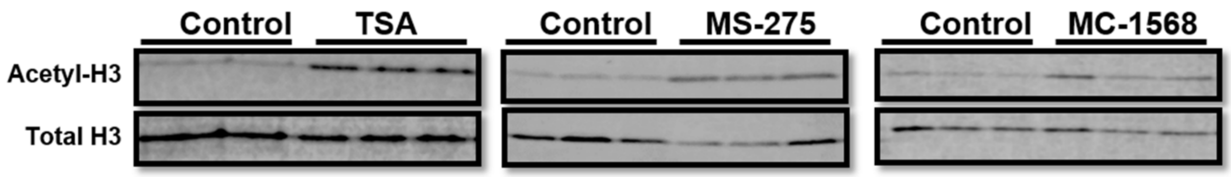

Supplemental Figure 10. TSA, MS-275 and MC-1568 stimulates acetylation of histone H3 in HIB1B cells. HIB1B cells were treated with TSA, MS-275 and MC-1568 as indicated, and acetyl-H3 and total H3 were analyzed via immunoblotting. n=3.
